# An Overview of Gastroduodenal Perforation

**DOI:** 10.3389/fsurg.2020.573901

**Published:** 2020-11-09

**Authors:** Elroy Patrick Weledji

**Affiliations:** Department of Surgery, Faculty of Health Sciences, University of Buea, Buea, Cameroon

**Keywords:** gastric, duodenum, perforation, etiology, Management, Operative, non-operative

## Abstract

Gastroduodenal perforation may be spontaneous or traumatic and the majority of spontaneous perforation is due to peptic ulcer disease. Improved medical management of peptic ulceration has reduced the incidence of perforation, but still remains a common cause of peritonitis. The classic sub-diaphragmatic air on chest x-ray may be absent and computed tomography scan is a more sensitive investigation in the stable patient. The management of perforated peptic ulcer disease is still a subject of debate. The majority of perforated peptic ulcers are caused by *Helicobacter pylori*, so definitive surgery is not usually required. Perforated peptic ulcer is an indication for operation in nearly all cases except when the patient is asymptomatic or unfit for surgery. However, non-operative management has a significant incidence of intra-abdominal abscesses and sepsis. Primary closure is achievable in traumatic perforation, but the management follows the Advanced Trauma Life Support (ATLS) principles.

## Introduction

Gastrointestinal perforation, with leakage of alimentary contents into the peritoneal cavity, is a common surgical emergency and may have life-threatening sequelae. Gastric perforation may be spontaneous or traumatic. The causes are listed in Table 1. The majority is from spontaneous perforation due to peptic ulcer disease (PUD) although there are more unusual causes (1, 2). The two main factors implicated in the etiology are non-steroidal anti-inflammatory drugs (NSAIDS) and *Helicobacter pylori* (*H. pylori)* (3, 4). Other factors include smoking, chronic liver disease, chronic renal failure, especially during dialysis and transplantation, and hyperparathyroidism. The incidence of peptic ulcer disease (PUD) is estimated to be _~_ 1.5–3%, the lifetime prevalence of perforation is _~_5% and mortality ranges from 1.3 to 25% (5). Below the age of 40 years duodenal ulcers are four times more common than gastric ulcers and are more common in men. Benign gastric ulcers occur predominantly in the elderly, on the lesser curve. Ulcers on the greater curve, fundus and in the antrum are more commonly malignant (5–8). Although <1% of gastric ulcers is pre-malignant, the percentage of cancer in gastric perforation (9%) is fairly significant (7). The surgical treatment with a simple omental patch closure of the perforation has not changed much over a century and PPU still remains a life-threatening condition with a high mortality of up to 40% being reported (8). Despite improvements in resuscitation techniques, antibiotic therapy and anesthesia, the mortality associated with perforated peptic ulcers over the last two decades remains about 25%. This is due to the fact that the age mix of the disease has changed with more elderly females on NSAIDs and many with serious concomitant medical illnesses (poor American Society of Anaesthesiologists score—ASA) (9). With the younger population in sub-Saharan Africa, the high mortality of PPU (~20%) is mostly due to the high prevalence of the causative *H. pylori* and late presentation (10–14).

**Table 1:** Causes of gastric perforation.

Spontaneous	Peptic ulceration Perforated carcinoma Gastric volvulus Strangulated hiatus hernia Ischemic disorders
Traumatic	Surgery Endoscopic/PEG complications Ventricul operitoneal (VP) shunt VP shunt complication Sharp foreign body Erosion by battery Stabwound Blunt abdominal trauma (rare)

## Spontaneous Perforation

Duodenal and gastric ulcers remain the two most common perforations of the gastrointestinal tract due to the increased use of NSAIDS. Acute ulcers along the anterior part of first part of duodenum usually perforate, whereas those on posterior aspect tend to cause bleeding as they erode into gastroduodenal artery. The lifetime risk of benign gastroduodenal perforation is 10% in untreated PUD and, 30–50% of ulcer perforations are associated with NSAIDS (1, 2). It occurs most often in elderly patients with co-existent medical problems, who are at increased risk of post-operative complications. The frequency of peptic ulcer and its perforation may change depending on the frequency of *H. pylori* infection and/or age distribution. The prevalence of *H. pylori* in the low socioeconomic classes and associated poverty, overcrowding, and poor hygiene have increased the incidence of duodenal and gastric perforations in all age groups particularly in the developing world (10–14). The mean prevalence of *H. pylori* infection in patients with perforated peptic ulcer is of only about 65–70%, which contrasts with the almost 90–100% reported in non-complicated ulcer disease. In addition, despite anti-ulcer medication and *H. pylori* eradication, perforated peptic ulcer (PPU) is still the most common indication for emergency gastric surgery, and associated with high morbidity and mortality. This might indicate that there are more factors involved in the pathology. This is also corroborated by the fact that only a third of patients with PPU had a history of peptic ulcer (4). Recurrent ulcer disease after peptic ulcer perforation, however, mainly occurs in patients with *H. pylori* infection which suggests its importance in this complication (4, 15). Perforated peptic ulcer is an important differential diagnosis to consider in patients with acute abdominal pain, but it only represents _~_3% of this group of patients (6–8). Sixty-seven percent of perforations were located in the duodenum and only 17% were gastric ulcers and, the specific diagnosis is usually only made at laparotomy (5). Most ulcers that perforate are on the anterior wall of the duodenum or stomach. The release of food and digestive enzymes into the peritoneal cavity initially causes a chemical peritonitis. Secondary bacterial peritonitis evolves later, and as with bleeding ulcers 10% of these patients will die (1, 2, 16). The presentation of gastric perforation is sudden onset severe epigastric pain, peritonism, a board-like abdominal rigidity caused by spasm of the recti muscles and sepsis, but may be non-specific in the elderly. The perforation is usually unexpected, with no antecedent history of PUD. The peritonitis is associated with varying degrees of shock, and severe peritonitis may induce a generalized ileus (17, 18). When posterior wall gastric ulcers perforate, they leak gastric contents into the lesser sac which tends to confine the peritonitis and present with less marked symptoms. There are some instances where patients do not have abdominal symptoms or signs, but chest x-rays taken for other reasons indicate a pneumoperitoneum. Perforated peptic ulcer is a common cause as the perforation is frequently sealed by a plug of omentum or another viscus before significant soiling and peritonitis occurs (18, 19). It is important to note that in this era of effective treatment of PUD with *H. pylori* eradication and proton pump inhibitors (PPIs), gastric cancer is commonly a cause of gastric outlet obstruction and perforation as opposed to peptic ulcer disease (20). Gastric volvulus and strangulated hiatus hernia (21) can lead to perforation if all or part of the stomach wall is rendered ischemic. Although the stomach has a good blood supply, on occasions severe foregut ischemia can lead to gastric ischemia and perforation, although such patients are generally unwell before the perforation is manifest (22). If perforation is in the thorax as in the case of strangulated hiatus hernia (HH), then the patient is likely to have chest symptoms and general signs of severe sepsis, with little or no evidence of peritonitis (21).

### Radiological and Laboratory Investigations

Radiological investigation form the basis of diagnosis. In the acute setting, an erect chest x-ray is invaluable as it not only often allows the diagnosis of pneumoperitoneum to be made with confidence but also gives information on the patient's general health, e.g., cardiomegaly, aspiration pneumonia, pulmonary metastases. A plain abdominal x-ray will demonstrate the double wall appearance of the intestines (Rigler's sign), a clear liver edge and air under the diaphragm “football sign” in the standing A–P view. When chest x-ray does not show pneumoperitoneum, or a relatively well-patient with a sealed perforation and uncertain diagnosis, a contrast enhanced computed tomography scan (CECT) of the abdomen is useful (19) as it has a high diagnostic accuracy of 98% (23). It will demonstrate pneumoperitoneum, pneumatosis intestinalis (intramural bowel gas) suggestive for necrotizing enterocolitis, perihepatic free fluid, air pockets around the stomach and thick reactive intestinal walls. The site of the perforation is sometimes visible as a region of discontinuity in the stomach or duodenal wall (7, 19). Pneumoperitoneum on erect chest x-ray is absent in 20–30% of cases, and if there is generalized peritonitis the diagnosis is confirmed at laparotomy or laparoscopy. Laboratory tests are performed in PPU not to establish the diagnosis but to rule out important differential diagnosis such as acute pancreatitis which has a similar presentation but different management and, to understand the insult on various organ systems such as renal function. Serum amylase may be raised in PPU but not to the level diagnostic of acute pancreatitis which is usually >4 times the upper limit of normal (i.e., >1,000 IU/L^−1^) (24).

## Management

The management of PPU may be operative or non-operative. The contributory factors to either of these are the general condition of the patient, poor pre-morbid status, significant co-morbidities, and complicated pathology (2, 17, 18). Most cases are within the remit of the general surgeon, but perforation due to strangulated hiatus hernia in chest is best dealt with by a dedicated upper gastrointestinal or thoracic surgeon.

### Operative Management

#### Perforated Duodenal Ulcer

**Figure 1:**
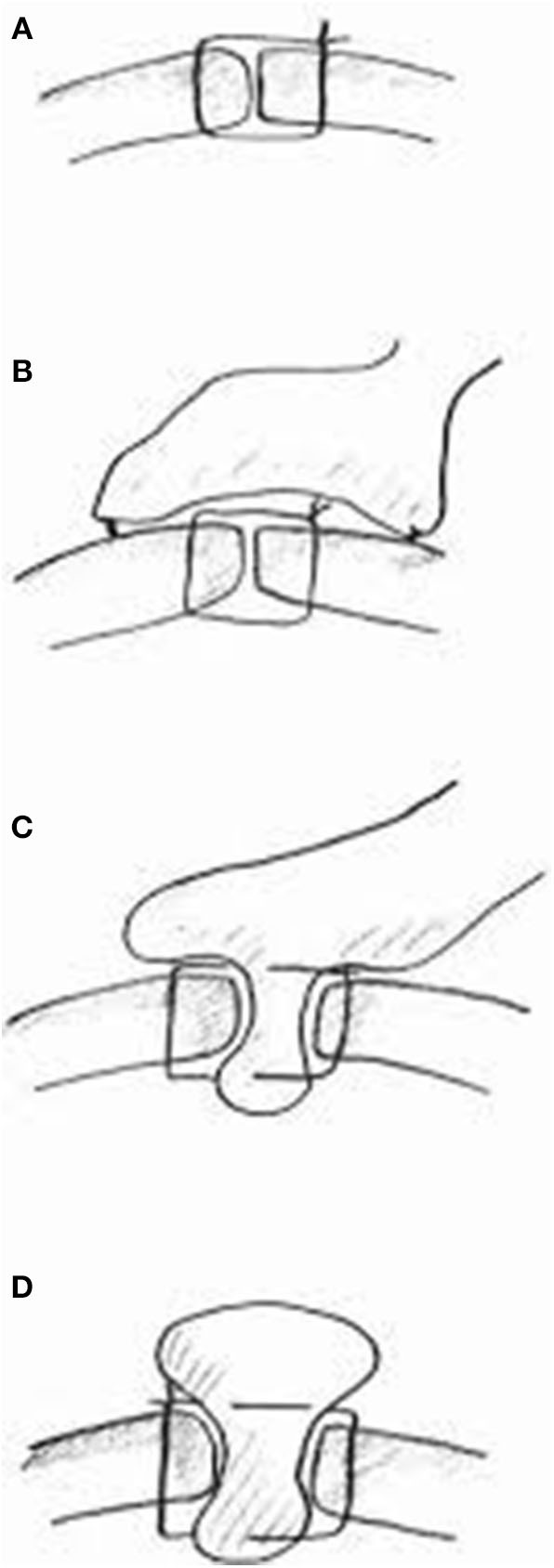
Summary of different suture techniques for closure of perforation [From above: **(A)** primary closure by interrupted sutures, **(B)** primary closure by interrupted sutures covered with pedicled omentopexy, **(C)** Cellan-Jones repair-plugging the perforation with pedicled omentoplasty, **(D)** Graham patch-plugging the perforation with free omental plug; with permission Bertleff and Lange (1)].

**Figure 2:**
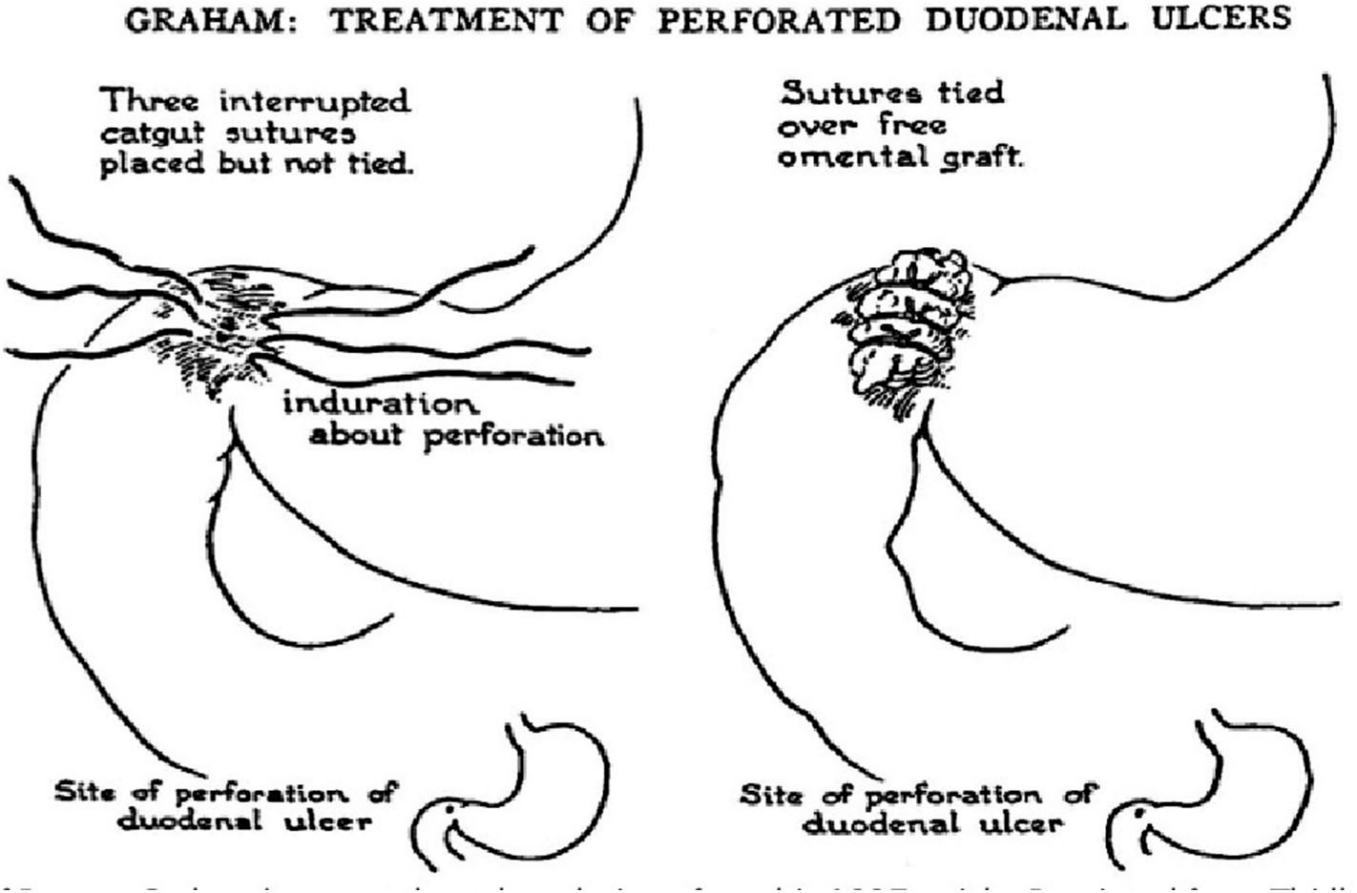
Schematic diagram of omental (Graham) patch technique (plugging with non-pedicle omental flap) [with permission: Graham (29)].

**Figure 3:**
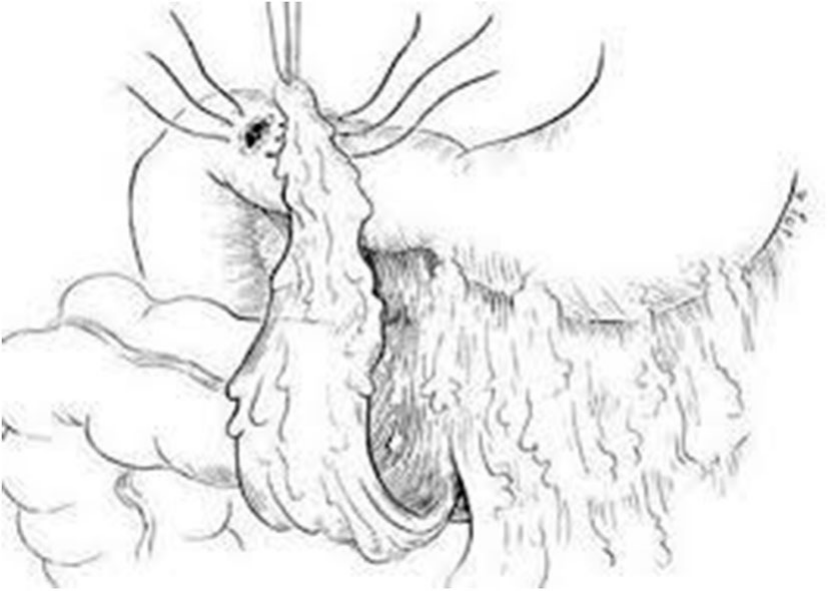
Schematic diagram of pedicled omental flap repair of perforated duodenal ulcer.

**Figure 4:**
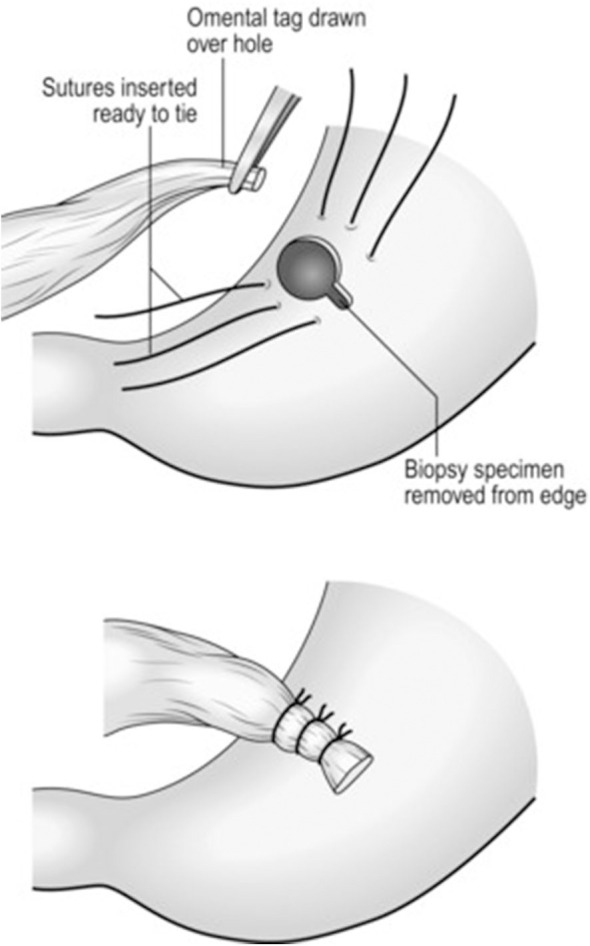
Schematic diagram of pedicled omental flap repair of gastric perforation.

The majority of perforated peptic ulcers are caused by *H. pylori*, so definitive surgery is not always required. With the advent of proton pump inhibitors (PPIs) and known peptic ulcer association with *H*. *pylori*, definitive ulcer preventing operations, i.e., vagotomy or gastrectomy, have largely been abandoned (25). However, definitive anti-ulcer surgery (parietal cell vagotomy ± anterior linear gastrectomy) can be performed for a perforated chronic duodenal ulcer previously shown to be *H. pylori* negative or those with recurrent ulcers despite triple therapy (1, 4, 5, 7, 11). The principle of operative management is to achieve a quick and easy access via a formal midline laparotomy and identify the site and nature of the pathology (25, 26). Suctioning of the gastrointestinal spillage and of any fibrinous exudates is quickly performed. This is facilitated by insinuating a hand between viscera and abdominal wall to make a space in which the sucker may be inserted, and both subphrenic spaces, the pericolic gutters and the pelvis are dealt with in turn. Attention is turned to inspection of the duodenum and visualization of the perforation. Improving access to the site of the perforation is aided by retracting the right margin of the incision and the assistant drawing the stomach and pylorus to the left by traction with a gauze swab. The perforation is usually found on the anterior wall in proximity to the duodenal bulb. If the perforation is not apparent, mobilization of the duodenum along with inspection of the stomach and jejunum is carried out. Most peptic ulcer perforations are small and easily closed (Figures 1A–C). The integrity of the repair may be confirmed by the “tire test' (air insufflation via the nasogastric (NG) tube. The simplest method which has amply stood the test of time is to plug the defect with a convenient frond of omentum which provides the stimulus for fibrin formation and tissue regeneration (Figure 1C) (27). Cellan-Jones in 1929 (28) suggested omentoplasty without primary closure of the defect to prevent narrowing of the duodenum. His technique consisted of placing 4–6 sutures, selecting a long omental strand and passing a fine suture through it. The tip of the strand is then anchored in the region of the perforation and finally the sutures are tied off (Figure 1C). In 1937 Graham (29) published his results with a free omental graft. He placed three sutures with a piece of free omentum laid over these sutures, which are then tied but no attempt is made to actually close the perforation (Figures 1D, 2). Very often surgeons mention using a Graham patch, but they actually used the pedicled omental patch described by Cellan-Jones which has since been the standard of surgical repair (Figures 1C, 3, 4) (30). The pedicled omental patch (Graham omentoplasty) technique entails passing through all layers of the duodenal wall using 0/0 or 2/0 absorbable vicryl on an atraumatic 30 mm needle, sufficiently far from the margin of the perforation to avoid tearing out because of friability. More than three such stitches are seldom necessary and in a small perforation, two may suffice. After placement, the sutures are left long and may be left in the tip of an artery forceps. A convenient fond of omentum with enough bulk to plug the defect is grasped in the tip of an artery forceps and drawn over the perforation to be held in place by the assistant. The stitches are then tied over the omental plug with just sufficient tension to retain the omental plug snugly in position. The top and bottom stitches being tied first so that tension in the middle stitch which is the most likely to cut out is reduced (Figures 1C, 3, 4) (28). Simple closure of the perforation by primary suture, then loosely suturing the omental flap over the closure with the ends of the primary suture (modified Graham patch repair/omentopexy) is the preferred method of dealing with perforation of <5 mm diameter (Figure 1B) (25, 26, 29, 31, 32). It is the first treatment of choice in early presentation of <12 h of PPU and when the patient is in shock (9, 19, 43) Recent studies in Africa continue to reveal that omentopexy still produces good results in patients with PPU (10–14). Graham's omentoplasty (plugging) and modified Graham's omental patch repair (omentopexy) are similarly effective repair in terms of morbidity and mortality (33–35). However, in several occasions with larger perforations the omental plugging seems a better choice to the omental patch reinforcement technique (omentopexy) (1, 34, 36, 37). A recent prospective study demonstrated a figure of eight primary closure with omental flap reinforcement to be more superior than Graham's omentoplasty (plugging) in terms of decrease leak rate in peptic perforations <2 cm in diameter (38).

### Dilemma of Duodenal Ulcer Perforation and Operative Hazards

The possible criticism that midline incisions are prone to dehiscence and herniation is answered by the use of the Jenkin's mass closure technique (39). Although operative management of a perforated duodenal ulcer (usually anterior D1) is generally straightforward, with an omental patch being fashioned after peritoneal lavage, Kocher's maneuver to mobilize the duodenum is performed if access to the duodenum is poor. Various methods are described to deal with this difficult duodenum (5, 40). Finney pyloroplasty involves fully Kocherizing the duodenum and opening it longitudinally along most of the length of the ulcer and then closed transversely in a similar fashion to simple pyloroplasty. A large perforation may lead to duodenum appearing to disintegrate and if it cannot be patched then it must be resected. More often, if the duodenal ulcer is too large and/or the tissues are too friable to perform an omental patch closure, a partial gastrectomy may be required. It may be necessary in some cases to exclude or excise the ulcer, close the duodenum distally, and excise the gastric antrum resulting to a Billroth II resection (40–42). If no perforation site is evident on initial laparotomy, the posterior surface of the stomach is exposed in the lesser sac. Infrequently perforation and hemorrhage from an anterior ulcer may coexist, and, partial gastrectomy of the Billroth II (Kronlein-Polya) type is advisable (16, 32, 41). *H. pylori* is the most important factor for ulcer recurrence following operative repair of perforated duodenal ulcer and merits eradication along with PPI therapy for about 4–6 weeks. Confirmation of eradication with Urea breath test is recommended in patients with resistant ulcer, MALT lymphoma and previous resection of gastric cancer (1, 4).

### Perforated Gastric Ulcer

A perforated gastric ulcer needs careful assessment. A proportion (9%) will be malignant (7) and gastric ulcers are more likely to re-perforate after simple closure with high mortality (15%) (6, 7). Tissue biopsies from the edge of the ulcer are taken because of the risk of malignancy, even in a benign-looking condition (1, 5, 43). The closure with an omental patch and *H. pylori* eradication as in duodenal perforation is feasible in distal or pre-pyloric ulceration as such ulcers are akin to duodenal ulcers (1, 5). Ulcer excision with post-operative PPIs, allows closure of ‘healthy’ gastric tissue, as well as providing histology, but, a distal gastrectomy with gastroduodenal anastomosis (Billroth I) should be considered if closure is difficult, the patient is sufficiently fit and the surgeon sufficiently experienced. Chung et al. (24) noted that <10% of PPU patients required gastric resection and with a mortality risk of 24 % the outcome was more inferior than omental patch repair. Follow-up endoscopy with repeat biopsy is still essential to avoid missing an underlying malignancy (1, 7, 24). In the pre-*H. pylori* eradication era, 80% of patients with simple omental closure alone developed recurrent ulcers. The mortality after surgery for PPU is between 6 and 19% (7, 10–14, 44). The four main factors which severely increase the mortality rate are (a) age>60 years, (b) delayed treatment (>24 h), (c) shock on admission (systolic BP <100 mmHg), and (d) concomitant diseases including HIV/AIDS (CD4 count <200 cells/μL) (10–14, 24, 45, 46). Gastric ulcers are associated with a two- to three-fold increased mortality risk (5, 47). Mortality is three- to four-fold higher in the elderly (up to 50%), due to occurrence of concomitant medical diseases and the difficulty in making the right diagnosis resulting in delayed treatment (6, 48). Factors such as shock on admission or delayed surgery were associated with omental patch leakage with increased mortality (49). The size of the opening may also determine the extent of the peritoneal contamination and adversely affects the prognosis. If the perforation is <5 mm in diameter there is a 6% mortality rate, when it is between 5 and 10 mm, the mortality is 19% and when it is more than 10 mm the mortality rate is around 24% (50). The choice of operative technique will depend on the position and size of the ulcer and the age and fitness of the patient. Perforated pre-pyloric ulcers are treated similarly to perforated DU, but more proximal gastric ulcers are best excised where possible. If it is likely to lead to significant stenosis then a patch repair can be performed (Figure 4). On some occasions it may be best to proceed with partial gastrectomy.

### Dilemma of Gastric Ulcer Perforation and Operative Hazards

Although the best palliation is resection of a perforated gastric tumor, at laparotomy the management is more difficult, especially with regard to decision-making in a critically-ill patient in whom speed and minimal tissue trauma is of over-riding importance (51). Even in cases of benign ulceration with perforation where tissue is edematous and swollen and have appearances of a neoplasm, decision to resect is difficult in these usually unstable patients. If any doubt as to how to proceed, immediate patient safety must come first, with peritoneal lavage and drainage as priority (41). Postoperative complications following repair of gastric ulcer perforation include intraperitoneal abscess in the subphrenic space or pelvis, persistence or recurrence of ulcer symptoms especially if post-operative *H. pylori* eradication was avoided, leakage from oversewn perforation, re-perforation, and gastric outlet obstruction from scarring of the duodenum (6).

### Is There a Role for Laparoscopic Surgery in Perforated Peptic Ulcer Disease?

Laparoscopic treatment of peptic ulcer perforation was first reported in 1990 (52) and suggested that laparoscopically performed omental patching was feasible and safe and had comparable results to open surgery with less postoperative discomfort (53–56). Laparoscopic repair using the easily mobilized falciform ligament for patch closure is a reasonable option in selected patients with a history of <24 h, no evidence of hypovolaemic shock, and with a perforation of <8–10 mm (57–59). Avoiding omentoplasty might shorten operating time but might be the reason for the higher incidence of leakage (60, 61). However, practice depends on expertise and local availability of laparoscopic surgery (8). The mortality after surgery despite technical and medical improvement was still 5.8% and the overall conversion rate for laparoscopic correction of perforated peptic ulcer was 12.4% (62). The diameter of perforation (often >1 cm), inadequate ulcer localization, and difficulties placing reliable sutures due to friable edges were the main reasons for conversion (62, 63). By using an omental patch a large perforation might not necessarily be a reason anymore to convert so long as the integrity of the repair is confirmed by the “tire test” (64). Other reasons associated with a significant conversion rate include failure to locate the perforation (21), shock on admission (50 vs. 8%) and time lapse between perforation and presentation (33 vs. 0%) (65). There is remarkable difference in morbidity (14.3%) in the laparoscopic group vs. (26%) in the open group, and mortality (3.55 vs. 6.4%) (65). A meta-analysis showed 85% success in the laparoscopic approach with reduced wound infection and pain (66). However, there was an increase rate of re-operation for leakage. This may be due to difficulty in the laparoscopic suturing procedure and the learning curve required (67). Thus, the need for a laparoscopically-trained surgeon to perform the procedure. Although the mortality and morbidity is comparable in other published series for open vs. laparoscopic approach, there has been no large randomized clinical trial comparing one against the other (65). Other methods include sutureless techniques involving the use of gelatin sponge plug with fibrin glue sealing or the use of endoscopic clipping techniques, but the complication and mortality rates are quite high limiting their use (68–71). Another minimally-invasive alternative is the insertion of self-expandable metal stents and drainage. This is one of the new treatment options for PPU which can be used primarily or secondarily to deal with post-operative leakage after surgical closure. A study involving 10 patients with PPU showed good clinical results (72). Following gastroduodenal perforation repair, peritoneal washout with several liters of warm saline would prevent inter-loop and intra-abdominal abscesses (73, 74). Although the outcome of laparoscopic closure of perforated peptic ulcer outweigh the disadvantages such as prolonged surgery time and greater expense, there is no consensus on whether it should be preferred over the open approach. Many trials are mostly non-randomized or retrospective. However, as laparoscopy can be both diagnostic and therapeutic for the acute abdomen (75), it should be advocated as a diagnostic and therapeutic tool in the case of suspected perforated peptic ulcer. Laparoscopic correction of PPU should be the first treatment of choice as it allows closure of the perforation and peritoneal lavage just like in open repair, but without a large upper abdominal incision (76). In addition, definitive ulcer surgery including posterior truncal vagotomy and anterior highly selective vagotomy is performed laparoscopically without conversion or mortality in expert hands (77). Nonetheless, it is not suitable for patients age over 70 years or for symptoms persisting longer than 24 h as there is associated morbidity and mortality (78).

### Pros and Cons of Drains

After a thorough wash out of the peritoneal cavity with 2–3 L of saline drainage of the peritoneal cavity is unnecessary. A routine drain insertion is unproven (79–82). A drain will not reduce the incidence of intra-abdominal fluid collections or abscesses (80). The drain site can become infected (10%) and the drain itself can cause intestinal obstruction (81). In case of suspected leakage, a CT scan will provide all the information needed, better than a non-productive drain (79–82). The evidence is that drains may cause more problems than they solve if they are placed ‘just in case’ of a leak. The adhesions that occur in the healing process of the repair, anastomosis, or general peritoneal cavity will attract the peritoneal drain (foreign body) which may physically damage the repair or small bowel. Secondly, the repair needs to gain some extra blood supply, which it does by forming adhesions to adjacent vascular structures. If a piece of corrugated plastic is placed beside a repair, it will be unable to do this and a leak will be encouraged. The only exceptions are where the repair is not watertight, such as bile or urine, and a collection will interfere with healing (79). There is a potential danger of suction (redivac) drains placed in the vicinity of a repair or anastomosis and, should be removed after 48 h (82). Drains can indeed mislead the surgeon as they easily get blocked. Large bore drains are useful in sepsis following inadequate peritoneal lavage or residual sepsis and should be placed in the appropriate dependent areas of the abdominal cavity such as the paracolic gutters, pelvis, and subphrenic spaces away from the intestine (82). Vigilance in the post-operative period is the key and to remember that leak can occur. Clinical signs backed by a water-soluble contrast study is the definitive investigation to determine if there is a leak (81).

### Non-operative Management

Most patients with perforated peptic ulcer should be treated by operation, but there is a small place for conservative management. Improvements in resuscitation techniques and the advent of powerful acid-suppressing agents (PPIs) have re-awakened interest in this treatment modality. The non-operative management is basically for (1) the asymptomatic and (2) the unfit patients. The asymptomatic patients are usually those who had typical symptoms of short duration with improvement by the time of hospital admission. Unlike gastric ulcer perforation, a large portion of duodenal ulcer perforation can be treated non-surgically (83). Pneumo-peritoneum has co-incidentally been discovered on erect chest or plain abdominal-x ray and, the computed tomography (CT) scan is used to investigate the pneumoperitoneum. The signs of peritoneal irritation are localized and when free gas is absent or minimal these patients have a small perforation which has already been sealed off with fibrin, omentum or an adjacent viscus. A conservative policy is appropriate if in addition to the above criteria, there is no antecedent dyspeptic history which is in favor of an acute rather than a chronic duodenal ulcer. Although in 1935, Wangensteen (1, 26, 84) reported a case series of 7 patients who recovered from perforated ulcers by self-healing, Herman Taylor in 1946 (26, 84) first reported 28 patients with perforated ulcers treated conservatively by nasogastric aspiration, intravenous (IV) fluids and serial abdominal X-rays (now known as Taylor's method) with a mortality of 10%. The efficiency of Taylor's method was established by Dascalescu et al. (84) who with the addition of broad spectrum antibiotics and anti-secretory drugs reported a success rate of 89%. Intra-abdominal abscess was the most common complication treated with antibiotics and drainage, but no mortalities. Early endoscopy is not advisable because of the risk of insufflation disrupting the plug which has sealed the gastroduodenal perforation, but it should be performed at a later stage to exclude malignancy. However, a definitive diagnosis is indispensable in performing non-surgical treatment because the perforation may lead to a fatal outcome. A water-soluble contrast meal may define those patients who do not have a free perforation into the peritoneal cavity or occasionally an endoscopic examination with carbon dioxide insufflation is useful (19–24, 84, 85). Free leakage of contrast medium into the peritoneal cavity is usually an indication for operative intervention (86). US/CT guided percutaneous drainage is an option for high risk patients who cannot tolerate major surgical treatment (87). Treatment with intravenous (IV) infusion, nasogastric tube (NGT) decompression, broad spectrum antibiotics, analgesia, and intravenous PPIs is instituted, and a nil by mouth (NBM) policy is initially adopted. Recovery is usually dramatically rapid for the properly selected patient and the right application of the protocol (88), but close observation is important as the development of sepsis or peritonitis may alter treatment radically, and CT-guided drainage may be required (9, 89–92). The mortality rate for non-operative management in patients with a sealed perforation was 3% as opposed to 6.2% where emergency surgery was performed for PPU (93). Small trials showed similar results to operative intervention and mortality rates of 5% in each group. Morbidity of 40% in the Taylor's method group vs. 50% in the surgical repair group has been reported in some studies (83). The exception was patients older than 70 years of age which was a factor associated with higher risk of surgical intervention. The study concluded that patients with perforated peptic ulcer may be observed in the initial 24 h and managed non-operatively (83). Thirty percent for whom non-operative treatment is initiated proceed to surgery, particularly if age is >70 (92). Other factors such as shock (hypotension) and comorbidities have also been described as factors contributing to the poor response to conservative approach and associated higher mortality (91). Thus, the decision of operative vs. conservative therapy depends on the patient's hemodynamic status and overall condition. Because of the significant incidence of intra-abdominal abscesses and sepsis with non-operative management, conservative management has been largely abandoned, even in high risk cases. This is encouraged by the current advances in anesthetic approach. Thus, non-operative treatment is advocated in selected patients who do not have generalized peritonitis or continued duodenal leak, and for those in whom there is an absolute contraindication for surgery. Nonetheless, it still has several problems: (1) the high rate of mortality as well as prolonged hospital stay in the case of treatment failure or misdiagnosis (5), (2) perforated gastric cancer is difficult to diagnose and will usually not respond, (3) gastric ulcer is less likely to respond to conservative therapy, but a large portion of duodenal ulcer perforation can, and (4) a colonic perforation is difficult to exclude and a free perforation will do badly with conservative treatment (90, 91). Non-operative management is less attractive in women than men because women who perforate are more likely to have a gastric than a duodenal ulcer (2, 7). The unfit patients are usually those with advanced peritonitis and sepsis with significant co-morbidity and/or poor pre-morbid function such as an acute myocardial infarction sustained a few days earlier, or an overwhelming pneumonia. They may be deemed unlikely to survive and it is important to discuss the implications with the patient and family. Perforation of an advanced gastric cancer may be another indication for pursuing a conservative course (91). In elderly patients with advanced cardiac or respiratory disease the benefit of the operation must be weighed against its hazards. In some of these patients, and in those who refuse operation non-operative management should be pursued with vigor and enthusiasm rather than a spirit of hopelessness.

### Perforated Stomal Ulcers

Perforated stomal ulcers are usually managed with omental patch (94). The usual anatomy will be distorted by the presence of either an antecolic, retrocolic gastroenterostomy or a Roux-en Y anastomosis. An antecolic gastroenterostomy is relatively easy to find as there will be a loop of small bowel anterior to the transverse colon to the stoma but a retrocolic gastroenterostomy may not be immediately apparent as it lies deep to the transverse colon and omentum.

### Perforated Hiatus Hernia/Gastric Volvulus

Perforated hiatus hernia or gastric volvulus, when part or all of the stomach is in the chest, present extremely difficult scenarios. Surgery in this situation may require thoracotomy, resection, and then a decision made regarding primary or delayed reconstruction (21, 22). The influencing factors are the time since presentation, degree of mediastinal and pleural soiling, and the general condition of the patient (21, 22, 95).

## Traumatic Perforation

Traumatic perforation follows major trauma. Gastric injury is suspected following penetrating or blunt abdominal injury (96). The management is along the lines of the Advanced Trauma Life Support (ATLS) principles in which injuries are managed in the order ABCDE: Airway, Breathing, Circulation, Disability (neurological injury) and Exposure, with priorities given to immediate life-threatening injuries (97). Gastric injury is likely to require surgery for hemorrhage and sepsis source control (98). It is vital to inspect carefully the anterior and posterior gastric wall, gastrooesophageal junction (GOJ), lesser sac entered with partial gastric mobilization, and to look for associated hepatic lacerations. Primary closure is generally feasible, but this is not possible in severe trauma where damage limitation surgery aimed at hemorrhage control and limiting the soiling of the peritoneum is of essence (99). Damage control surgery entails the acute resection (stapling-off) of damaged tissue, drainage and delayed reconstruction at re-look laparotomy at 48 h. This will allow the correction of physiology and avoid the lethal triad of death from hypothermia (temp < 34°C), coagulopathy (PT >16 s) and acidosis (pH < 7.2). Thus, the correction of physiology takes precedence over anatomical correction in the exsanguinating critically ill patient. It is important to remember that acute gastric dilatation although commonly seen in trauma, is a rare but important postoperative complication of major upper abdominal surgery, post-splenectomy and with the gastric autonomic neuropathy of diabetes mellitus and, may cause gastric perforation (100–102). From the author's experience, the subtle presentation of left shoulder tip pain and hiccups from diaphragmatic irritation may lead to it being unrecognized and untreated with a fatal outcome due to vomiting and aspiration. The correction of any biochemical abnormalities, such as potassium is essential, and the treatment is by large bore NG tube with regular aspiration (103).

## Conclusions

The majority of gastroduodenal perforations are spontaneous from peptic ulcer disease. The management is not standardized as it essentially depends on the clinical scenario and the surgeon's experience. Perforated peptic ulcer is an indication for operation in nearly all cases except when patient is unfit for surgery. Surgical techniques are varied, but laparotomy and omental patch repair remains the gold standard while laparoscopic surgery should only be considered when expertise is available. This must be followed by *H. pylori* eradication therapy to prevent recurrence. Gastrectomy is recommended in patients with large or malignant ulcer to enhance outcome. Primary closure is achievable in traumatic perforation but with the exsanguinating critically ill patient in severe major trauma, damage limitation surgery to correct physiology prior to a later anatomical reconstruction is the principle of management.

## Author Contributions

The author confirms being the sole contributor of this work and has approved it for publication.

## Conflict of Interest

The author declares that the research was conducted in the absence of any commercial or financial relationships that could be construed as a potential conflict of interest.
